# Prevalence and factors associated with caesarean delivery on maternal request and its effect on maternal and foetal outcomes in selected tertiary care hospital, Odisha, Southeastern India

**DOI:** 10.7189/jogh.15.04073

**Published:** 2025-03-21

**Authors:** Sindhu Singh, Dharitri Swain

**Affiliations:** College of Nursing, All India Institute of Medical Sciences (AIIMS), Bhubaneswar, Odisha, India

## Abstract

**Background:**

Caesarean delivery is now predominantly performed in response to the mother’s request, often without medical indications, commonly referred to as caesarean delivery on maternal request (CDMR). The rise in CDMR has become a significant issue in maternal and newborn health. We aimed to explore the factors influencing CDMR and its effect on maternal and foetal outcomes.

**Methods:**

We used a prospective cohort design approach to recruit 413 participants and a consecutive sampling technique to select the participants. Those who fulfilled the inclusion criteria were taken during the six-month data collection period from October 2023 to March 2024. We used a structured interview method for data collection. We utilised inferential statistics, such as Fisher exact and χ^2^ tests for univariate analysis and a logistic regression model in the multivariate analysis, to investigate the relationship between factors and mode of delivery.

**Results:**

The multivariate regression analysis revealed that the CDMR rate was higher among the women who preferred caesarean delivery before giving birth (odds ratio (OR) = 6.295; 95% confidence interval (CI) = 1.468–26.995, *P* < 0.05). Additionally, women with a history of previous caesarean delivery were more inclined to choose CDMR in the subsequent pregnancy (OR = 25.642; 95% CI = 1.199–548.221, *P* < 0.05). The likelihood of experiencing wound pain (OR = 42.374; 95% CI = 14.612–122.887, *P* < 0.05), encountering breastfeeding difficulties (OR = 11.469; 95% CI = 2.91–45.2, *P* < 0.05), and neonatal intensive care unit admissions (OR = 0.268; 95% CI = 0.076–0.95, *P* < 0.05) was significantly higher in CDMR compared to normal vaginal delivery.

**Conclusions:**

The prevalence of CDMR was 21.35%, which was relatively higher than the World Health Organization’s recommended guidelines. The previous mode of delivery and maternal preference for caesarean birth were the factors that influenced CDMR. It is necessary to make childbirth readiness counselling a regular practice to assist women in selecting the best delivery method.

Medically necessary caesarean deliveries are performed to protect high-risk pregnant women and their unborn children by preventing complications such as maternal haemorrhage, foetal discomfort, and other potentially fatal conditions. However, the adverse effects of caesarean delivery on maternal and foetal birth outcomes have progressively come to light as it is being performed without any medically indicated cases more frequently [1].

The global significant variations in the rate of caesarean delivery on maternal request (CDMR) can be observed across different countries. China exhibits a rate exceeding 24%, while Sweden reports a rate of 8%, Switzerland at 5.1%, Denmark at 3.2%, and the USA and Australia both at 3% across all the deliveries [2]. In Saudi Arabia, various studies have been conducted to investigate CDMR. However, only one study, which employed a retrospective cohort study design utilising medical records, reported a CDMR rate of 10.1% [2]. The World Health Organization (WHO) has released recent data showing that the number of caesarean deliveries performed globally is increasing and currently represents more than one in five (21%) deliveries. According to the report, the percentage is expected to rise over the next ten years, with nearly a third (29%) of all births expected to be by caesarean delivery by 2030 [3]. The WHO recommends a CDMR rate within the range of 10–15% [4]. In contrast, the prevalence of CDMR in India is significantly higher, ranging from 17.2–48%, depending on the specific region [5]. This alarming trend, which exceeds international guidelines, requires more research into the factors that motivate mothers to have caesarean deliveries.

Caesarean delivery is now predominantly performed in response to the mother’s request, commonly referred to as ‘maternal request.’ In 2007, the American College of Obstetricians and Gynaecologists defined CDMR as the primary caesarean delivery that is performed at the request of the mother without any medical or obstetric indication present [1,6]. This has emerged as the leading indication for opting for a caesarean delivery during childbirth. The main reason for caesarean delivery, which accounted for 23.38% of all caesarean deliveries, was maternal request reported by a study in China [1].

CDMR is a multifaceted concern involving ethical, medical, and social ramifications, necessitating a comprehensive understanding of its frequency, underlying drivers, and potential consequences. It has been shown by several research on various factors associated with CDMR, including anxiety, a traumatic birth experience, a lack of maternal awareness, and a lack of decision-making options by counselling on the benefits and harms of different modes of delivery [7].

Research indicates that the caesarean delivery procedure prevents about 187 000 maternal deaths and approximately 2.9 million neonatal deaths worldwide [8]. Although caesarean delivery significantly lowers maternal and neonatal mortality, non-medically advised caesarean deliveries may increase the risks to the mother’s and the child’s short and long-term health [8]. Long-term concerns include asthma and obesity, while short-term concerns include visceral injury, placenta accretion, placental abruption, postpartum haemorrhage, and infection [9,10]. Additionally, there is a higher chance of neonatal intensive care unit (NICU) admission and delayed breastfeeding with caesarean delivery [11].

Previous studies conducted in Eastern India, Odisha, have suggested that the incidence of caesarean delivery is associated with various personal factors experienced by women during pregnancy, as well as other external factors [12,13]. However, there is limited information available on why mothers choose to undergo caesarean birth without any medical indication. In this study, we aimed to assess the influencing factors of CDMR and to determine its adverse effect on maternal and foetal birth outcomes compared to normal vaginal delivery (NVD).

## METHODS

### Study design and setting

We conducted a hospital-based prospective cohort study to assess the prevalence and factors associated with CDMR and its effect on maternal and foetal outcomes in selected tertiary care hospitals in Odisha, Southeastern India, for six months from October 2023 to March 2024. The rationales for selecting the setting were the approval to conduct the research, the easy availability of subjects, and cooperation from the departments.

### Study participants and sample size

In this study, we included all third-trimester pregnant women who attended antenatal care at the selected hospital. Inclusion criteria were: 1) women in the third trimester of pregnancy who were going to delivery at the study centre, 2) women with singleton pregnancy, 3) women who had consented to participate, and 4) women who could understand and read Odia or English. Those who were not able to understand the questions and were mentally ill were excluded. The total sample size of 444 was calculated from the prevalence of CDMR cases from a previous research study with the probability (p) of exposure among controls p_0_ = 0.3601, the probability of exposure among cases p_1_ = 0.2338, [1] a significance level of 5% and a power of 80%. For the final analysis, we included 413 samples because 31 women did not deliver at the study centre. We used consecutive sampling to recruit the desired participants for the study.

### Ethical approval and consent to participate

Before data collection, we obtained ethical approval from the Institutional Ethics Committee and the concerned hospital authority. The investigator explained about the research purpose and duration of the interview to all the participants by providing a patient information sheet. We obtained informed written consent from all the participants for their voluntary participation in the study, and they had the right to withdraw from participation at any point during the data collection period. It was assured that confidentiality would be maintained and that data would not be used for any purpose other than research.

### Data collection and measurement tool

We collected all data in the obstetrics and gynaecology outpatient department and the postnatal ward. Data collection was carried out from October 2023 to March 2024. We collected data in two phases. We collected the first phase data during the third trimester of pregnancy during antenatal visits, and the second phase was collected in the postnatal period within seven days or before discharge to assess the factors associated with CDMR and its birth outcome. Pregnant women who consented to be study participants were recruited using a consecutive sampling technique. After obtaining written consent, the data collection of the first phase began using the structured interview method, where face-to-face interviews were conducted for 10–15 minutes. The second phase of data collection started after delivery, using birth outcome structured checklists. The researchers filled out the checklists by observing surgical site infection, chronic/wound pain through facial expressions, and breastfeeding difficulties. Also, we checked patient records for indications of caesarean delivery, mode of delivery (*e.g.* NVD, CDMR, *etc.*), and low appearance, pulse, grimace, activity, and respiration (APGAR) scores. We used a structured interview method to assess postpartum depression. The researchers interviewed each participant and checked the postnatal records to obtain data within seven days or before discharge. On average, six to 10 participants were interviewed each day. No incentive was provided to the participants to take part in the study.

We administered a structured and standardised tool to assess the different factors such as socio-demographic factors, obstetrical factors, maternal psychological factors, family factors, social factors, postnatal depression, and birth outcomes. We established the content validity and reliability of the tool before administration, and the same investigator was assigned to interview all participants to avoid the interpretation biases. We measured baseline characteristics by a semi-structured demographic and prenatal questionnaire. Socio-demographic factors included age, level of education, place of residence, occupation, and monthly income. Obstetrical factors included body mass index (BMI), smoking history, previous medical history, previous pregnancy complications, present pregnancy complications, gravidity, parity, previous mode of delivery, pregnancy and childbirth history, family history, exercise during pregnancy, antenatal education, prenatal visit, and preference of delivery mode.

We used the pregnancy pressure scale to assess the stress level experienced by women during the antenatal period. The questionnaire consisted of 24 items [14]. The score ranged from 0 to 72 and is scored as 0 (no stress), 1–24 (mild stress), 25–48 (moderate stress), and 49–72 (severe stress). Score for each response was based on the frequency Likert scale – zero (no pressure), one (mild pressure), two (moderate pressure), and three (severe pressure).

We used the family adaptation, partnership, growth, affection, and resolve scale was used to measure the family support care and consists of a family support questionnaire that includes 5 items [15]. The score ranges from 0–10, and scores from 0–3 refer to severe impairment in family functions, 4–6 refer to moderate impairment in family functions, and 7–10 refer to good family functions. The score for each response is based on a frequency Likert scale (zero meaning hardly ever, one meaning some of the time, and two meaning almost always).

We used the social support rating scale to measure social support received by women during pregnancy, and consists of a social support questionnaire that includes 9 items [16]. The score ranges from <35 to >45, with scores as <35 (low level), 35–45 (moderate level), and >45 (high level). In this particular scale, responses range from 1–4 for questions one to four and seven to nine, which are associated with one to four points, respectively. Regarding question five, according to the support degree of the a–d options, participants answered one to four to each option to obtain one to four points. In the case of question six, the numbers of support sources, ranging from one to nine, are exactly equal to the scores one could obtain.

We used the Edinburgh postnatal depression scale to measure postpartum depression, which is a standardised tool consisting of 10 items [17]. The Edinburgh postnatal depression scale ranges <8 (no depression), 9–11 (mild depression), 12–13 (moderate depression), and ≥14 (severe depression). In this scale, score ranges from 0–3 for questions one, two, and four, and the score range is 0–3 for questions three, and 5–10.

We checked the tool’s language validity for correctness before data collection. We then measured birth outcomes, such as maternal and newborn outcomes, using a structured birth outcome checklist.

### Statistical analysis

For all statistical analyses, we used SPSS, version 22.0 (IBM, Chicago, Illinois, USA). We summarised the characteristics of participants, and we used frequencies and percentages to describe the sociodemographic characteristics, obstetrical characteristics, family characteristics, social characteristics, and birth outcomes of pregnant women. We used inferential statistics, such as Fisher exact and χ^2^ tests, for univariate analysis. To investigate the relationship between these factors and the mode of delivery further, we employed a logistic regression model in the multivariate analysis. In this analysis, the mode of delivery was considered the dependent variable, with NVD coded as ‘0’ and CDMR coded as ‘1’. We selected variables in the univariate analysis (*P* < 0.1) as independent variables. Using professional knowledge and information from the literature, we included gravidity and pregnancy and childbirth history in the adjustment model [1].

## RESULTS

### Demographics and maternal characteristics

In demographic characteristics, the majority, 94 (49.0%), of participants were in the age group of 25–30 years. A considerable portion, 113 (58.9%), resided in urban areas. A higher education predominated among the participants, 110 (57.3%). A significant proportion of participants, 153 (79.7%), were unemployed/housewives. Monthly income was predominantly concentrated in the middle range, with 87 (45.3%) earning between INR 15 001–55 000. Concerning maternal characteristics, the majority, 142 (74.0%), had a BMI≥24. Most of the women, 157 (81.8%), did not experience complications from previous pregnancies, whereas 48 (25.0%) reported complications from their current pregnancies. Regarding gravidity, 105 (54.7%) were primigravida, while 127 (66.2%) were nulliparous, 2 (1.0%) had a history of stillbirths, 40 (20.8%) reported a previous history of abortions, and 150 (78.1%) reported no adverse pregnancy outcomes. Previous modes of delivery show that 34 (17.7%) had normal vaginal deliveries, while 29 (15.1%) had a caesarean delivery, and 129 (67.2%) were primigravida and had no childbirth history). When it comes to preferences for delivery mode for the present delivery, 101 (52.6%), preferred vaginal delivery, while 43 (22.4%) had no clear preference, and 48 (25.0%) favoured caesarean sections (**Table 1**).

**Table 1:** Demographics and maternal characteristics of participants (n = 192)

Characteristics	n (%)
Total	192 (100)
Age in years	
*<25*	54 (28.1)
*25–30*	94 (49.0)
*>30*	44 (22.9)
Place of residence	
*Urban*	113 (58.9)
*Rural*	79 (41.2)
Level of education	
*No formal education*	1 (0.5)
*Primary school*	5 (2.6)
*Junior high school*	31 (16.2)
*Secondary education*	45 (23.4)
*Higher studies*	110 (57.3)
Occupation	
*Unemployed/housewives*	153 (79.7)
*Employed*	39 (20.3)
Monthly income in INR	
*≤15 000*	80 (41.6)
*15 001–55 000*	87 (45.3)
*≥55 001*	25 (13.0)
BMI in kg/m^2^	
*<18.5*	4 (2.0)
*18.5–23.9*	46 (24.0)
*≥24*	142 (74)
Previous pregnancy compilation	
*No*	157 (81.8)
*Yes*	35 (18.2)
Present pregnancy compilation	
*No*	144 (75.0)
*Yes*	48 (25.0)
Gravidity	
*Primigravida*	105 (54.7)
*Multigravida*	87 (45.3)
Parity	
*0*	127 (66.2)
*≥1*	65 (33.9)
Pregnancy and childbirth history	
*Stillbirth*	2 (1.0)
*Abortion*	40 (20.8)
*No bad history*	150 (78.1)
Previous mode of delivery	
*Vaginal*	34 (17.7)
*Caesarean section*	29 (15.1)
*Other (primigravida and no childbirth history)*	129 (67.2)
Preference of delivery mode	
*No clear preference*	43 (22.4)
*Vaginal delivery*	101 (52.6)
*Caesarean section*	48 (25.0)

BMI – body mass index

### Prevalence and factors associated with CDMR

**Figure 1:**
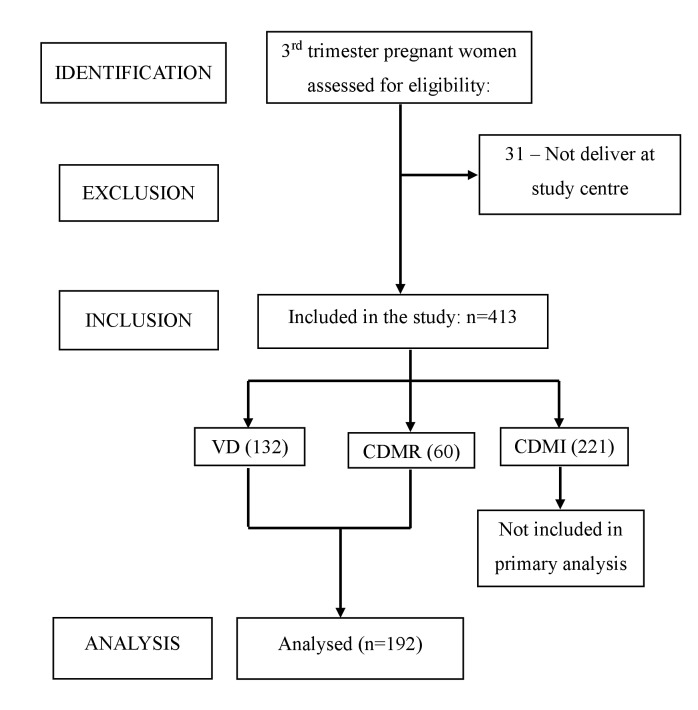
Flow diagram of the study participant as per STROBE guidelines. CDMI – caesarean delivery on medical indication, CDMR – caesarean delivery on maternal request, VD – vaginal delivery.

Out of the 413 participants enrolled in the study, 132 had undergone NVD, and 281 had caesarean delivery. Of the 281 caesarean deliveries, 221 were caesarean deliveries on medical indication, and 60 were CDMR. Based on the objective, we included 132 NVD and 60 CDMR in the analysis. To assess the prevalence of CDMR and its birth outcomes, the delivery mode of the participants was recorded from the hospital record (**Figure 1**).

In the univariate analysis, socio-demographic factors, including maternal age, occupation, and socio-economic status, were associated with CDMR (*P* < 0.05) (**Table 2**). Obstetrical factors, including BMI, previous medical history, previous pregnancy complications, poor pregnancy and childbirth history, previous mode of delivery, the preference of delivery mode, and maternal psychological factors such as perceived stress during the antenatal period were associated with CDMR (*P* < 0.05) (**Table 3**). Family and social factors included advice from husband, parents, parents-in-law, delivery mode of friends, friends, and doctor’s advice and were also found to be associated with CDMR (*P* < 0.05) (Table S1 in the **Online Supplementary Document**).

**Table 2:** Association between socio-demographic factors with CDMR (n = 192)

Items	NVD, n (%)	CDMR, n (%)	Df	χ^2^/FE	*P-*value
Total	132 (100.00)	60 (100.00)			
Age			2	11.263	0.004
*<25*	46 (34.85)	8 (13.33)			
*25–30*	62 (46.97)	32 (53.34)			
*>30*	24 (18.18)	20 (33.33)			
Place of residence			1	2.2	0.13
*Urban*	73 (55.30)	40 (66.67)			
*Rural*	59 (44.70)	20 (33.33)			
Level of education			4	2.493*	0.71
*No formal education*	1 (0.76)	0 (0.00)			
*Primary school*	3 (2.27)	2 (3.33)			
*Junior high school*	24 (18.18)	7 (11.67)			
*Secondary education*	32 (24.24)	13 (21.67)			
*Higher education*	72 (54.54)	38 (63.33)			
Occupation			1	9.141	0.002
*Unemployed/ housewives*	113 (85.61)	40 (66.67)			
*Employed*	19 (14.39)	20 (33.33)			
Monthly income in INR			2	16.067	<0.001
*≤15 000*	55 (41.67)	25 (41.67)			
*15 001–55 000*	68 (51.51)	19 (31.67)			
*≥55 001*	9 (6.82)	16 (26.66)			

CDMR – caesarean delivery on maternal request, Df – degrees of freedom, FE – Fisher exact test, NVD – normal vaginal delivery

*Fisher exact test.

**Table 3:** Association between obstetrical factors with CDMR (n = 192)

Items	NVD, n (%)	CDMR, n (%)	Df	χ^2^/FE	*P-*value
Total	132 (100.00)	60 (100.00)			
BMI			2	10.263*	0.004
*<18.5*	3 (2.27)	1 (1.67)			
*18.5–23.9*	40 (30.30)	6 (10.00)			
*≥24*	89 (67.42)	53 (88.33)			
Smoking history			2		0.55
*No*	129 (97.73)	60 (100.00)			
*Active*	0 (0.00)	0 (0.00)			
*Passive*	3 (2.27)	0 (0.00)			
Previous medical history			1	4.176	0.04
*No*	118 (89.39)	47 (78.33)			
*Yes*	14 (10.61)	13 (21.67)			
Previous pregnancy complication			1	23.664	<0.001
*No*	120 (90.91)	37 (61.67)			
*Yes*	12 (9.09)	23 (38.33)			
Present pregnancy complication			1	0.129	0.71
*No*	100 (75.76)	44 (73.33)			
*Yes*	32 (24.24)	16 (26.67)			
Gravidity			1	2.954	0.086
*Primigravida*	77 (58.33)	27 (45.00)			
*Multigravida*	55 (41.67)	33 (55.00)			
Parity			1	2.379	0.12
*0*	92 (69.70)	35 (58.33)			
*≥1*	40 (30.30)	25 (41.67)			
Pregnancy and childbirth history			2	4.569*	0.081
*Still birth*	2 (0.15)	0 (0.00)			
*Abortion*	22 (16.67)	18 (30.00)			
*No bad history*	108 (7.58)	42 (70.00)			
Previous mode of delivery			2	42.380*	<0.001
*Vaginal delivery*	32 (24.24)	2 (3.33)			
*Caesarean section*	6 (4.55)	23 (38.33)			
*Other*	94 (71.21)	35 (58.34)			
Family history			1	0.077	0.78
*No*	101 (76.52)	47 (78.33)			
*Yes*	31 (23.48)	13 (21.67)			
Exercise during pregnancy			1		1
*No*	6 (4.55)	3 (5.00)			
*Yes*	126 (95.45)	57 (95.00)			
Received antenatal education			1	0.429	0.51
*No*	55 (41.67)	22 (36.67)			
*Yes*	77 (58.33)	38 (63.33)			
Number of prenatal visits			3	5.749	0.12
*<6*	67 (50.76)	23 (38.33)			
*6–10*	47 (35.61)	22 (36.67)			
*11–15*	14 (10.60)	9 (15.00)			
*>15*	4 (3.03)	6 (10.00)			
Preference of delivery mode			2	56.445	<0.001
*No clear preference*	30 (22.73)	13 (21.67)			
*Vaginal delivery*	89 (67.42)	12 (20.00)			
*Caesarean section*	13 (9.85)	35 (58.33)			
Maternal psychological factors			3	15.907	<0.001
*No stress*	55 (41.67)	09 (15.00)			
*Mild stress*	75 (56.82)	47 (78.33)			
*Moderate stress*	2 (1.51)	04 (06.67)			

CDMR – caesarean delivery on maternal request, Df – degrees of freedom, FE – Fisher exact test, NVD – normal vaginal delivery

*Fisher exact test.

We performed a logistic regression model to determine the predictors of CDMR, which was statistically significant, χ^2^ 29 = 111.939), *P* < 0.001. The model explained Nagelkerke *R2 =* 62% of the variance in CDMR and correctly classified 85.9% of CDMR development. The results of the multivariate analysis revealed that individuals with a history of previous caesarean delivery were 25 times more likely to choose caesarean delivery in the subsequent pregnancy, compared to normal vaginal delivery (odds ratio (OR) = 25.642; 95% confidence interval (CI) = 1.199–548.221, *P* = 0.03). Additionally, women who expressed a preference for caesarean delivery prior to giving birth were six times more likely to undergo CDMR in comparison to those without a clear choice of delivery mode (OR = 6.295; 95% CI = 1.468–26.995, *P* = 0.013) (Table S2 in the **Online Supplementary Document**).

### Relationship between CDMR and maternal and foetal outcome

The odds of experiencing chronic/wound pain (OR = 42.374; 95% CI = 14.612–122.887, *P* < 0.05) were 42.37 times higher in CDMR compared to NVD. In foetal birth outcomes, the likelihood of encountering breastfeeding difficulties (OR = 11.469; 95% CI = 2.91–45.2, *P* < 0.05) was 11.47 times higher, and NICU admissions (OR = 0.268; 95% CI = 0.076–0.95, *P* < 0.05) were 0.27 times higher in CDMR compared to NVD (Table S3 in the **Online Supplementary Document**).

## DISCUSSION

CDMR is a significant health care issue that requires improvement in multiple areas, including clinical decision-making, health care policy formulation, and research. In India and other nations, addressing CDMR involves evaluating its prevalence and understanding the socio-cultural and psychological factors that influence maternal request. Healthcare professionals must enlighten expectant mothers and dispel any misconceptions regarding caesarean delivery during prenatal visits.

In our study, by exploring CDMR through univariate analysis, we identified sociodemographic characteristics, age, occupation, and monthly income as factors associated with the decision-making process regarding the mode of delivery. These findings emphasise the importance of considering broader socio-economic factors in understanding women’s preferences and choices surrounding childbirth. Notably, similar findings have been identified in Chinese, Armenian, Bangladeshi, and Kenyan studies, which showed an association with age, occupation, and monthly income [18–22]. In our study, various obstetric factors emerged as significant factors associated with CDMR. These included BMI, previous medical history, previous pregnancy complications, previous history of caesarean delivery, preference of caesarean delivery mode, and maternal psychological factors. These findings are supported by a body of literature from China, Ethiopia, and South Africa, which has shown that factors such as BMI index, previous medical history, childbirth history, and preference for caesarean delivery mode were associated with CDMR [1,18,23–25]. Similar factors have been identified in the other Indian study, including BMI, previous pregnancy complications, and history of caesarean delivery, as being associated with CDMR [26].

In our study, we examined the factors associated with CDMR, and significant associations have been identified with various family dynamics. Specifically, the husband’s advice, parent’s advice, and advice from parents-in-laws have emerged as influential factors shaping the decision-making process regarding delivery mode. Similar findings have been observed in some other studies conducted in countries like China, Ghana, and Uganda, where the impact of advice from the husband, parent, and parents-in-law influences the mother’s decision to go for caesarean delivery [1,27,28]. In certain African cultures, the responsibility for making decisions regarding women's pregnancy and childbirth is often entrusted to husbands and mothers-in-law within the family [28].

In our study, social factors such as the delivery mode of friends and family, friend's advice, and doctor’s advice were associated with CDMR. A study conducted in China reported similar findings, where the delivery mode of friends and family, friends’ advice, and doctors’ advice were associated with CDMR [1]. Additionally, similar findings were observed in other studies conducted in Brazil and Uganda as well where friends’ advice was associated with CDMR [7,27].

We revealed a significant association between the previous mode of caesarean delivery and CDMR, and it was found that previous caesarean posed a 25 times higher risk for having CDMR compared to NVD. This trend was consistent with the findings from previous literature from the Netherlands, Nepal, Saudi Arabia, and Egypt, where an increased probability of caesarean delivery was reported due to previous caesarean delivery [29–32]. We revealed that the rate of CDMR was higher among women who chose caesarean delivery during late pregnancy without medical indications. Similar findings were observed in other studies from China, and the USA, where an increased incidence of CDMR was observed among women who chose caesarean delivery during late pregnancy without medical necessity [1,33]. A study from the Netherlands indicates women who preferred NVD but ended up with caesarean delivery are at risk of experiencing severe fear of childbirth postpartum [29].

### Birth outcomes associated with CDMR

In our study, postpartum depression and other complications, such as chronic/wound pain and breastfeeding difficulties, have been identified as essential determinants in the context of caesarean delivery. Similar findings were observed in a study conducted in Japan, where postpartum depression was associated with caesarean birth [34]. Similar findings were observed in other studies from China, the USA, and Canada, where low APGAR scores [11] and breastfeeding difficulties [35,36] were associated with caesarean delivery.

In our study, it reveals that there was a substantial increase, approximately 42.37 times, in the chances of mothers developing chronic or wound pain after undergoing CDMR. Another study based on global data also supports this claim by highlighting the potential link between caesarean delivery and chronic pain [8]. Furthermore, an increased risk of encountering breastfeeding challenges was observed to be 11.47 times more likely in women who underwent CDMR. Research findings also indicate a potential delay in breastfeeding initiation associated with caesarean deliveries, as highlighted in previous studies from Canada [8,36]. Additionally, there was a notable statistical association between NICU admissions and cases of CDMR. Similar findings were observed in other studies from Saudi Arabia, Canada, and the USA where NICU admissions were associated with caesarean deliveries [31,36,37]. The Canadian study also indicates NICU admission was the most common neonatal outcome observed in both planned CDMR and planned NVD [36].

Our study has several strengths, including the use of a hospital-based prospective cohort study design, and inclusion of various maternal, family and social factors associated with CDMR. The consecutive method was used for data collection, and data was collected from all the women who had come for antenatal visits in their third trimester of pregnancy. However, certain limitations are acknowledged here. It is a single-centre study with a small sample size as a limitation, so the results may not be generalised to the population of other settings in India. The data was collected from pregnant women during the third trimester of pregnancy due to the limitation of the study duration, which might not entirely reflect the impact of the entire pregnancy status on the delivery outcome. Some information, especially postpartum depression, was assessed within the 7 days or before the discharge of the patient, which can be followed up to 6 weeks of postpartum for diagnosis of depression.

### Recommendations

Based on the present study, we recommend preconception counselling and antenatal education as essential to address the rise in CDMR. Providing accurate information about the risks and benefits of different delivery methods can help mothers make informed choices. Additionally, fostering open communication between nurses, midwives, and expectant mothers can facilitate better understanding and support for the best delivery options tailored to individual circumstances. Nurses and midwives’ practitioners must encourage evidence-based practices and implement delivery guidelines in maternity care that can contribute to more balanced decision-making regarding delivery methods. Addressing the issue requires a multidisciplinary approach involving health care providers, policymakers, and community education to ensure informed decision-making.

## CONCLUSIONS

In this study, we showed that the occurrence of CDMR is related to the previous mode of delivery and the preference for delivery mode before childbirth. Additionally, the mother may experience postoperative chronic or wound-related pain. On the other hand, CDMR can also increase the risk of breastfeeding difficulties and NICU admission compared to NVD. Healthcare professionals must educate women about the associated risks of CDMR and encourage discussions that address both the benefits and possible complications of the procedure. Lastly, there is a need to develop a policy for the mode of delivery and protocol to avoid unnecessary surgical procedures, prolonged hospital stays, and pocket expenditures.

## Additional material


Online Supplementary Document
