# Supplementary material for: Prevalence and factors associated with caesarean delivery on maternal request and its effect on maternal and foetal outcomes in selected tertiary care hospital, Odisha, Southeastern India
Source: J Glob Health. 2025 Mar 21;15:04073. doi: 10.7189/jogh.15.04073 (PMC11927758; doi:10.7189/jogh.15.04073)
Supplement: Online Supplementary Document [file jogh-15-04073-s001.pdf]

Supplement to: Singh S, Swain D. Prevalence and factors associated with caesarean delivery on maternal request and its effect on maternal and foetal outcomes in selected tertiary care hospital, Odisha, Southeastern India. J Glob Health. 2025;15:04073.

Table S1. Association between family and social factor with CDMR (n=192)

	**NVD**	**CDMR**			
**Factors**	**f (%)**	**f (%)**	**df**	**χ2/ FE**	***P* value**
Total	132 (100)	60 (100)			
**I. Family Factors**					
Husband's advice			2	FE=21.695	< 0.001
No recommendation	78 (59.09)	46 (76.67)			
Recommend VD	52 (39.39)	7 (11.67)			
Recommend CD	2 (1.52)	7 (11.66)			
Parent's advice			2	FE=18.976	< 0.001
No recommendation	76 (57.58)	44 (73.33)			
Recommend VD	54 (40.91)	9 (15.00)			
Recommend CD	2 (1.51)	7 (11.67)			
Advice from parents-in-law			2	FE=17.006	< 0.001
No recommendation	77 (58.33)	45 (75.00)			
Recommend VD	53 (40.15)	9 (15.00)			
Recommend CD	2 (16.67)	6 (10.00)			
Family Support			2	FE=2.234	0.28
Low	2 (1.52)	1 (1.67)			
Medium	24 (18.18)	6 (10)			
High	106 (80.30)	53 (88.33)			
**II. Social Factors**					
Medical staff service			2	FE=1.070	0.72
Good	120 (90.91)	53 (88.33)			
General	11 (8.33)	7 (11.67)			
Poor	1 (0.76)	0 (0.00)			
Delivery mode of friends, family			2	17.144	< 0.001
VD	104 (78.79)	32 (53.33)			
CD	8 (6.06)	15 (25.00)			
The two delivery methods are equal	20 (15.15)	13 (21.67)			
Friend's advice			2	FE=9.988	0.004
No recommendation	95 (71.97)	49 (81.67)			

Recommend VD	37 (28.03)	8 (13.33)			
Recommend CD	0 (0.00)	3 (5.00)			
Doctor's advice			2	FE=7.271	0.019
No recommendation	120 (90.91)	55 (91.67)			
Recommend VD	12 (9.09)	2 (3.33)			
Recommend CD	0 (0.00)	3 (5.00)			
Social Support			2	FE=0.167	1
Low	10 (7.58)	5 (8.33)			
Moderate	117 (88.63)	53 (88.34)			
High	5 (3.79)	2 (3.33)			

$\chi 2$ - Chi-Square test, CD - caesarean delivery, CDMR - caesarean delivery on maternal request, df - degrees of freedom, FE - Fisher's exact test, NVD - normal vaginal delivery, VD - vaginal delivery.

Table S2. Multivariate logistic regression analysis for predictors of CDMR (n=192)

**Factors**	**β**	**Wald**	**OR**	**95% CI**		***P* value**
				**Lower**	**Upper**	
Previous mode of delivery						
Vaginal delivery						reference
Caesarean section	3.244	4.311	25.642	1.199	548.221	0.03
No previous delivery	0.395	0.08	1.484	0.096	22.969	0.77
Preference of delivery mode						
No clear preference						reference
Vaginal delivery	-1.177	2.657	0.308	0.075	1.269	0.1
Caesarean section	1.84	6.133	6.295	1.468	26.995	0.013

β - regression coefficient, CI - confidence interval, NICU - neonatal intensive care unit, OR - odds ratio.

Table S3. Multivariate logistic regression analysis of birth outcome with CDMR (n=192)

**Characteristics**	**β**	**Wald**	**OR**	**95% CI**		***P* value**
				**Lower**	**Upper**	
**I. Maternal birth outcomes**						
Any other (chronic/wound pain)						
No						reference

Yes	3.747	47.565	42.374	14.612	122.887	< 0.001
**II. Neonatal birth outcomes**						
Breast feeding difficulties						
No						reference
Yes	2.44	12.157	11.469	2.91	45.2	0.001
NICU admission						
No						reference
Yes	-1.315	4.159	0.268	0.076	0.95	0.04

β - regression coefficient, CI - confidence interval, NICU - neonatal intensive care unit, OR - odds ratio.
